# Chasing the High, Losing the Beat: A Case of Cocaine-Induced Myocardial Infarction

**DOI:** 10.7759/cureus.81158

**Published:** 2025-03-25

**Authors:** Saurabh Kumar Singh, Chirag Agrawal, Anbhigya Kumar Arya, Satyajit Padhiary, Devesh Kumar

**Affiliations:** 1 Cardiology, Vardhman Mahavir Medical College and Safdarjung Hospital, New Delhi, IND

**Keywords:** acute myocardial infarction, cardiovascular complications, chest pain, first-time cocaine use, young male

## Abstract

Cocaine use is a well-established risk factor for various cardiovascular complications, including acute myocardial infarction (MI). The pathophysiological mechanisms responsible for cocaine-induced MI are multifactorial, encompassing coronary vasoconstriction, increased myocardial oxygen demand, and thrombosis. While cocaine-related cardiovascular events are more commonly seen in individuals with pre-existing risk factors, cases involving young, otherwise healthy individuals remain rare, yet they present a significant concern. We present the case of a young male in his late teens who presented to the emergency department with acute-onset chest pain that began two hours following his first-time use of cocaine. On evaluation, it was an anterior wall MI. Urgent coronary angiography was performed, which showed an ostial cut-off in the left anterior descending artery, along with a Thrombolysis in Myocardial Infarction (TIMI) grade V thrombus. Percutaneous coronary intervention (PCI) was successfully performed, and TIMI III flow was achieved. He was discharged on day five post-procedure, and at a six-month follow-up, he was doing well. This case highlights the need for early recognition and prompt intervention, including coronary angiography and PCI, to improve outcomes. Furthermore, it emphasizes the potential for long-term success with proper follow-up care and adherence to prescribed therapies.

## Introduction

Cocaine use is an ominous problem among all races and ethnicities. The largest age group at risk for cocaine use is adults between 18 and 25 years of age. Cocaine is one of the most commonly used illicit drugs in patients seeking care in emergency departments, and it is one of the most frequent causes of drug-related deaths [1]. Cocaine has been associated with multi-system involvement, and myocardial infarction (MI) and cerebrovascular accidents have a temporal relationship with cocaine use. Cocaine-related cardiac manifestations include MI, aortic dissection, arrhythmias, myocardial dysfunction, and Takotsubo cardiomyopathy. Chest pain is the chief complaint among cocaine users, and a pragmatic approach is warranted for the evaluation of these patients to rule out MI/aortic dissection.

## Case presentation

A young man in his late teens presented with complaints of acute onset of severe, retrosternal chest pain radiating to both arms for two hours after the first-time use of cocaine. It was associated with two episodes of loss of consciousness before he could reach the emergency department. He had no significant family history of cardiac illness or premature atherosclerotic cardiovascular disease. On examination, his blood pressure was 170/120 mmHg and he had tachycardia at a heart rate of 120 beats/minute. The rest of his examination was within normal limits.

**Figure 1:**
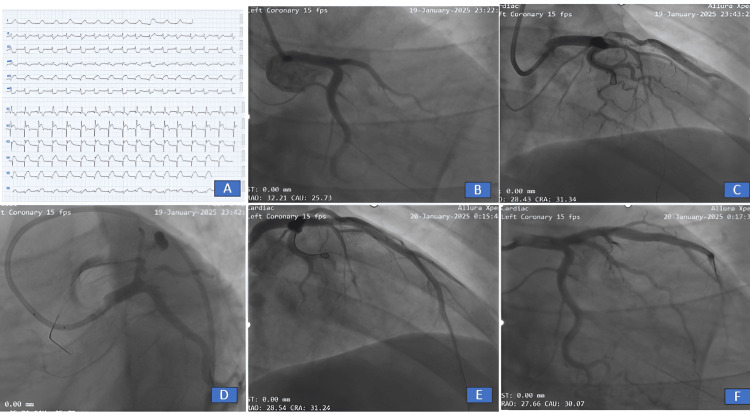
(A) A standard 12-lead electrocardiography showing anterolateral wall myocardial infarction (QRBBB with ST elevation in chest leads V1-V6 and I, AVL with reciprocal ST depression in Il/lIl/AVF). (B) Coronary angiography right anterior oblique caudal view showing the left anterior descending artery 100% cut-off from the ostia. (C and D) After balloon dilatation flow achieved with thrombus. (E and F) Final flow achieved post-stenting in right anterior oblique cranial and right anterior oblique caudal view, respectively.

**Figure 2:**
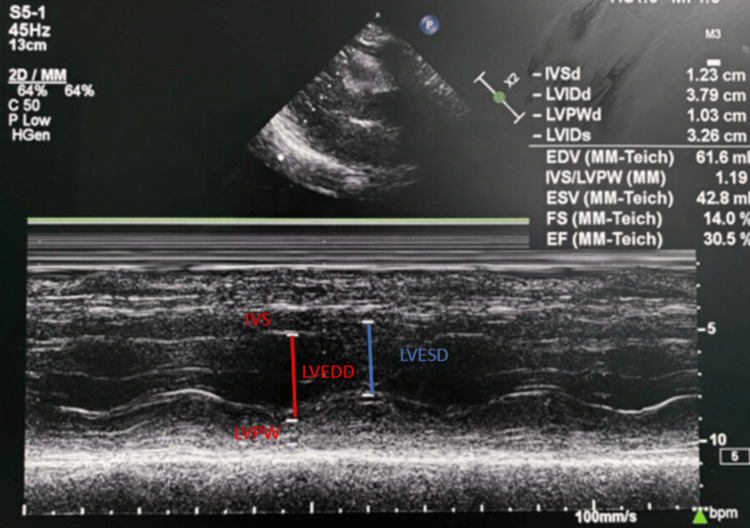
Transthoracic M-mode echocardiography from parasternal long-axis view showing a left ventricular ejection fraction of 30%. IVS = interventricular septum; LVPW = left ventricular posterior wall; LVEDD = left ventricular end-diastolic diameter; LVESD = left ventricular end-systolic diameter

His 12-lead electrocardiography revealed a normal axis, normal sinus rhythm, qRBBB pattern with ST elevation in the chest leads V1-V6 and I, AVL with reciprocal ST depression in II/III/AVF, suggestive of anterolateral wall MI (Figure 1, Panel A). Two-dimensional transthoracic echocardiography revealed anterior, anteroseptal, and anterolateral wall hypokinesia with a left ventricular ejection fraction of 30% (Figure 2, Video 1). Routine blood investigations including renal function tests, liver function tests, and a hemogram were within normal limits. The enzyme-linked immunosorbent assay for human immunodeficiency virus (HIV) and syphilis was negative. Lipid profile revealed total cholesterol of 120 mg/dL, serum triglycerides of 90 mg/dL, high-density lipoprotein of 44 mg/dL, and low-density lipoprotein of 60 mg/dL. Serum homocysteine level was within normal limits at 9 µmol/L (5-15 µmol/L). Inflammatory markers such as erythrocyte sedimentation rate and C-reactive protein were within normal limits. Antinuclear antibody (ANA) and antiphospholipid antibody (APLA) profiles were negative. A workup for hypercoagulability in the form of protein C, protein S, anti-thrombin-III, and factor V Leiden mutation was negative (Table 1).

**Video 1:** Transthoracic echocardiography short-axis view at the level of the papillary muscle showing anterior, anteroseptal, and anterolateral wall hypokinesia.

**Table 1:** Laboratory investigations. TLC = total leukocyte count; AST = aspartate aminotransferase; alanine aminotransferase; ALP = alkaline phosphatase; HIV = human immunodeficiency virus; VDRL = Venereal Disease Research Laboratory; HDL = high-density lipoprotein; LDL = low-density lipoprotein; ESR = erythrocyte sedimentation rate; CRP = C-reactive protein; ANA = antinuclear antibody; APLA = antiphospholipid antibody

Parameters	Patient values	Reference values
Hemoglobin (g/dL)	15.8	11.5–17
TLC (cells/mm^3^)	15,100	4,000–11,000
Platelets (L/mm^3^)	2.7	1.5–4.5
Urea (mg/dL)	32	17–43
Creatinine (mg/dL)	0.9	0.6–1.3
AST (U/L)	146	10–35
ALT (U/L)	651	10–45
ALP (U/L)	124	40–128
Total bilirubin (mg/dL)	1.4	0.3–1.2
Sodium (mmol/L)	139	136–145
Potassium (mmol/L)	4.1	3.5–5.5
HIV I and II	Negative	
VDRL	Negative	
Lipid profile		
Total cholesterol (mg/dL)	120	<200
Triglyceride (mg/dL)	90	<150
HDL (mg/dL)	44	40–60
LDL (mg/dL)	60	<100
Hypercoagulable state		
Homocysteine (µmol/L)	9	5–15
Protein C	Negative	
Protein S	Negative	
Anti-thrombin-III	Negative	
Factor V Leiden mutation	Negative	
Inflammatory and autoimmune screening		
ESR	14	<15
CRP	Negative	
ANA	Negative	
APLA	Negative	

Our patient presented with signs and symptoms consistent with acute coronary syndrome and, on evaluation, was found to have an anterolateral wall myocardial infarction (ALWMI). Considering his young age, we evaluated all possible etiologies of acute coronary syndrome in a young patient. There were no cutaneous lesions suggestive of xanthomas, no family history of premature coronary artery disease, no features suggestive of familial hypercholesterolemia, and no features suggestive of connective tissue disorders or endocrinopathy. Secondary hypercoagulable states such as hyperhomocysteinemia, protein C, protein S, anti-thrombin-III, and factor V Leiden mutation were ruled out. An autoimmune screen using ANA and APLA profiles was negative. There was no evidence of polycythemia or malignancy. HIV and syphilis were also carefully excluded.

Given ALWMI within the window period (two hours), he was taken up for urgent coronary angiography. Right femoral arterial access was taken, and coronary angiography was suggestive of 100% thrombotic ostial cut-off (Figure 1, Panel B), with other vessels left circumflex artery and the right coronary artery being normal. Percutaneous coronary intervention (PCI) to the left anterior descending (LAD) artery was subsequently planned. The lesion in LAD was crossed using a workhorse wire and subsequently dilated with a semi-compliant 2.5 × 15 balloon (Figure 1, Panels C, D). After balloon dilatation, the patient developed 3 episodes of ventricular fibrillation, which were reverted with DC cardioversion. Intracoronary tenecteplase 10 mg and injection abciximab 10 mg were given for Thrombolysis in Myocardial Infarction (TIMI) grade V thrombus. A drug-eluting stent (Xience Xpedition) (4 × 29 mm) was deployed at 14 ATM for 20 seconds, and TIMI III flow was achieved (Figure 1, Panels E, F). The procedure was uneventful, and the patient was shifted to the coronary care unit and kept on injection abciximab infusion for 24 hours.

## Discussion

Cocaine, derived from the *Erythroxylum coca* plant, is the second most commonly used illicit drug in the United States and is responsible for the highest number of emergency department visits related to drug use [1]. The global prevalence of cocaine abuse presents a significant challenge due to its severe physical, psychological, and social consequences. Cocaine exists in two primary forms, namely, powder cocaine (cocaine hydrochloride) and crack cocaine, a smokable form that is associated with an increased risk of cardiovascular events, particularly MI [2]. The onset and duration of cocaine’s effects vary depending on the route of administration, leading to different cardiovascular and hemodynamic responses [3]. Intravenous and inhaled cocaine have a rapid onset of action (seconds) and a short duration (approximately 30 minutes) compared to mucosal routes (oral, nasal, rectal, vaginal), which produce a more prolonged effect.

Upon administration, cocaine inhibits the presynaptic reuptake of norepinephrine and dopamine, causing an accumulation of catecholamines at the postsynaptic receptors and acting as a potent sympathomimetic agent [4]. This increases heart rate and blood pressure in a dose-dependent manner, significantly elevating cardiac workload and oxygen demand [5]. Additionally, cocaine induces coronary vasoconstriction, which is more pronounced in diseased arterial segments, contributing to ischemia. The vasoconstrictive effect is likely mediated through the stimulation of alpha-adrenergic receptors on smooth muscle cells in coronary arteries, and it also sensitizes vascular smooth muscle to catecholamines, compounding vasoconstriction beyond receptor stimulation alone. While the use of phentolamine and verapamil is mechanistically logical, their routine clinical use in the emergency setting is not standard or widely practiced. Evidence is limited, and use is more theoretical or selective, not mainstream guideline-based practice [6,7]. Cocaine also alters the balance of vasoconstrictors and vasodilators by increasing endothelin-1 (a potent vasoconstrictor) and decreasing nitric oxide (a vasodilator) levels, further promoting vasoconstriction [8]. This combination of increased oxygen demand and reduced blood supply leads to ischemic events associated with cocaine use. Furthermore, cocaine enhances platelet activation, aggregation, and an increase in platelet count, further contributing to thrombus formation and the development of MI [9]. Autopsy studies in young cocaine users have shown evidence of coronary atherosclerosis with associated thrombus formation, indicating that cocaine may precipitate premature coronary artery disease and thrombosis [10].

The typical demographic of cocaine users includes young men, many of whom smoke cigarettes, a factor that compounds the cardiovascular effects of cocaine use. In these individuals, the combined effect of cocaine and smoking results in a greater increase in heart rate and vasoconstriction than either substance alone [7].

Chest pain is the most common presenting symptom in cocaine users presenting to emergency departments, occurring in up to 50% of cases of cocaine-induced MI. This pain is often described as pressure-like in quality [11]. Other symptoms may include dyspnea, anxiety, palpitations, dizziness, and nausea. The risk of MI is significantly increased, up to 24-fold, during the first hour after cocaine use, with the highest risk observed in first-time users and those using the intranasal route [12]. Cocaine-associated MI typically occurs within three hours of use, though it can manifest anywhere from one minute to four days after ingestion. In the COCHPA study, only 6% of patients with chest pain after cocaine use developed an MI. Overall, the prevalence of MI among patients presenting with chest pain following cocaine use is reported to be between 0.6% and 6%, with a low mortality rate (0.09%) in this cohort, suggesting that patients without ST-segment elevation can be safely observed in the emergency department, potentially reducing unnecessary admissions and improving resource utilization [13].

A systematic approach to evaluating patients with cocaine-associated chest pain is critical and includes obtaining a detailed history, conducting a thorough physical examination, and monitoring vital signs. A 12-lead electrocardiogram and measurement of cardiac troponins are essential components of the diagnostic workup. Stable patients should be observed for at least 12 hours. The management of cocaine-induced MI mirrors that of non-drug-related MI, with some key exceptions. Aspirin and clopidogrel are recommended to address the increased platelet aggregation and coronary thrombosis [14]. Cocaine-induced coronary vasoconstriction can be reversed with nitrates, phentolamine (an alpha-receptor blocker), and verapamil (a calcium channel blocker). According to the American College of Cardiology/American Heart Association guidelines for ST-segment elevation MI, beta-blockers should not be administered to patients with cocaine-induced ST-elevation MI, as they may exacerbate coronary spasms [12]. In these cases, PCI to restore blood flow is preferred over fibrinolysis, though fibrinolytic therapy should be considered if PCI is unavailable. However, fibrinolysis is associated with a higher risk of intracranial bleeding in cocaine-related MI and should only be used when PCI is not an option. Data are limited and mostly anecdotal or case-based.

Other cardiac manifestations of cocaine use include myocardial dysfunction, arrhythmias (such as atrial fibrillation and ventricular arrhythmias such as ventricular extrasystoles, ventricular tachycardia, and ventricular fibrillation), aortic dissections, and Takotsubo cardiomyopathy [15]. Takotsubo cardiomyopathy, also known as stress cardiomyopathy, is usually triggered by intense emotional or physical stress and occurs in relatively older age groups with female preponderance. Cocaine use is also linked to an increased risk of ischemic stroke in young adults.

Cessation of cocaine use is the most critical step in secondary prevention. Recurrent chest pain, MI, and death are less common in patients who discontinue cocaine use. Despite the significant cardiovascular risks associated with cocaine, it remains a public health concern, necessitating ongoing efforts in prevention, treatment, and research. A multifaceted approach involving healthcare providers, policymakers, and communities is essential to mitigate the impact of cocaine on individual and societal health. In conclusion, cocaine-induced MI represents a serious condition that affects a group of young individuals, and these patients may face recurrent complications following their initial presentation.

## Conclusions

Cocaine use should be considered a potential cause of MI, particularly in younger individuals who do not present with conventional cardiovascular risk factors. In such cases, the possibility of drug-induced MI must be promptly evaluated, and clinicians should consider cocaine use as a primary etiology when diagnosing ST-elevation MI. Early recognition and accurate diagnosis are essential for the appropriate management of these patients. PCI should be prioritized over thrombolytic therapy for cocaine-induced ST-elevation MI, as it offers more effective and immediate restoration of coronary blood flow. Pharmacological management must be approached with caution, particularly concerning avoiding beta-blockers, as these may exacerbate coronary vasospasm. Instead, treatments that reduce vasoconstriction and sympathetic stimulation, such as nitrates, alpha-blockers, and calcium channel blockers, should be used to alleviate the effects of cocaine-induced coronary spasms. Finally, addressing the underlying cause of the event, i.e., cocaine addiction, is critical for long-term recovery and the prevention of future cardiovascular complications. A comprehensive approach to treatment, including addiction management, is necessary to reduce the risk of recurrence and to ensure better overall health outcomes for these patients. This multifaceted strategy can significantly improve both the immediate and long-term health of individuals affected by cocaine-related MI.
